# Regeneration of the dentin-pulp complex in vital pulp therapy: biological basis, biomaterials, and clinical translation

**DOI:** 10.3389/fdmed.2026.1857656

**Published:** 2026-06-25

**Authors:** Ayako Washio

**Affiliations:** Division of Endodontics and Restorative Dentistry, Department of Regenerative Science in Conservative Dentistry, Kyushu Dental University, Kitakyushu, Fukuoka, Japan

**Keywords:** biomaterial, cell, dentin-pulp complex, growth factor, regenerative endodontics, scaffold, translational research, vital pulp therapy

## Abstract

Vital pulp therapy has traditionally aimed to preserve pulp vitality; however, conventional approaches primarily induce reparative dentin formation rather than true regeneration of the dentin-pulp complex. Advances in regenerative medicine have shifted this paradigm toward biologically driven reconstruction targeting both structural and functional restoration. This mini-review provides a translational overview of dentin-pulp complex regeneration, integrating stem/progenitor cell biology, angiogenesis, neurogenesis, immune modulation, growth factor signaling, and biomaterial-based strategies. Particular emphasis is placed on distinguishing repair from functional regeneration and conceptualizing regeneration as a biological continuum. Recent advances in bioactive scaffolds, controlled-release systems, and three-dimensional biofabrication are highlighted as key to regulating the regenerative microenvironment. Biologically stratified approaches based on pulpal condition and residual tissue viability are discussed to improve predictability. Although emerging studies support the feasibility of regenerative strategies, direct comparative evidence with conventional vital pulp therapy remains limited, and current outcome measures are not fully aligned with functional restoration. Standardized evaluation criteria and integration with preventive care are essential for advancing clinical translation.

## 1 Introduction

Vital pulp therapy (VPT) has traditionally been performed as a conservative approach to preserve pulp vitality following caries removal or traumatic injury (1). The objective of VPT has been to preserve pulp vitality and avoid invasive procedures such as root canal treatment. Conventional VPT procedures, including indirect pulp capping, direct pulp capping, and pulpotomy, primarily rely on the formation of tertiary dentin as a protective barrier against external stimuli (2).

However, tertiary dentin formation is generally considered a reparative response rather than regeneration. Tertiary dentin is often structurally irregular and does not fully restore dentin-pulp (D-P) complex architecture or function (3). Consequently, although conventional VPT can achieve acceptable clinical outcomes in terms of symptom relief and pulp survival, it falls short of achieving full biological restoration.

Recent advances in regenerative medicine have significantly shifted the paradigm of VPT toward biologically driven regeneration (4, 5). This shift has been facilitated by developments in stem cell biology, biomaterials, and signaling molecule delivery systems. Experimental studies have demonstrated that pulp-like tissue containing vascular and neural components, as well as dentin-like structures with varying degrees of structural organization, can be regenerated under controlled conditions (6–8). These findings suggest that functional regeneration is a realistic therapeutic goal. To clarify the terminology used throughout this review, “repair” refers to mineralized tissue formation without restoration of the original D-P architecture or biological function. “Partial regeneration” indicates incomplete reconstruction of specific tissue components, whereas “functional regeneration” refers to restoration of both structural and biological characteristics of the D-P complex. This distinction is important because mineralized tissue formation and pulp survival alone do not necessarily indicate restoration of native D-P structure or function (9).

Among emerging strategies, scaffold-based tissue engineering has gained particular attention (10). Scaffolds not only provide a structural framework but also act as a microenvironment that supports cell adhesion, proliferation, and differentiation. Notably, studies have demonstrated that bioactive scaffolds incorporating bioactive glass and growth factors enhance tissue organization and reduce inflammatory responses, highlighting their translational potential (11). Furthermore, controlled-release systems have enabled sustained delivery of growth factors such as fibroblast growth factor-2 (FGF-2), improving regenerative outcomes (12, 13). Advanced biofabrication techniques, including three-dimensional (3D) bioprinting, allow precise spatial control of cell placement, which is essential for reconstructing the hierarchical structure of the D-P complex.

**Figure 1:**
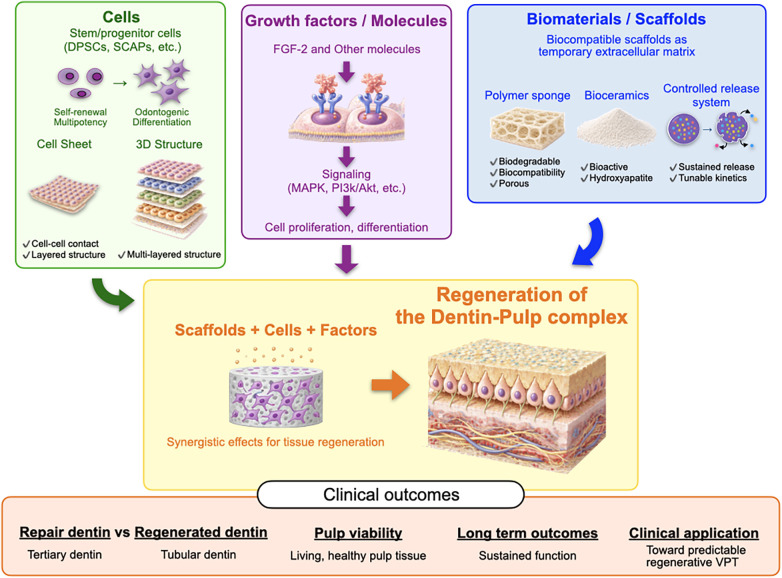
Integrated strategy for dentin-pulp complex regeneration based on cells, signaling molecules, and biomaterials. This schematic illustrates a tissue engineering-based approach for dentin-pulp complex regeneration through the integration of stem/progenitor cells, growth factors, and biomaterial scaffolds. Stem/progenitor cells, including dental pulp stem cells (DPSCs) and stem cells from the apical papilla (SCAPs), possess self-renewal capacity, multipotency, and odontogenic differentiation potential. Cell sheet technology and three-dimensional (3D) constructs enhance cell-cell interactions and promote tissue organization. Growth factors, such as fibroblast growth factor-2 (FGF-2), regulate cellular behaviors via intracellular signaling pathways (e.g., MAPK and PI3 K/Akt). Biomaterial scaffolds, including biodegradable polymer sponges and bioactive ceramics, function as a temporary extracellular matrix, providing structural support and biological cues. Controlled-release systems enable sustained delivery of signaling molecules. The synergistic interaction of cells, signaling molecules, and scaffolds promotes regeneration of the dentin-pulp complex, aiming to restore biologically functional tissue characterized by tubular dentin formation, maintenance of pulp vitality, and long-term functional stability.

**Figure 2:**
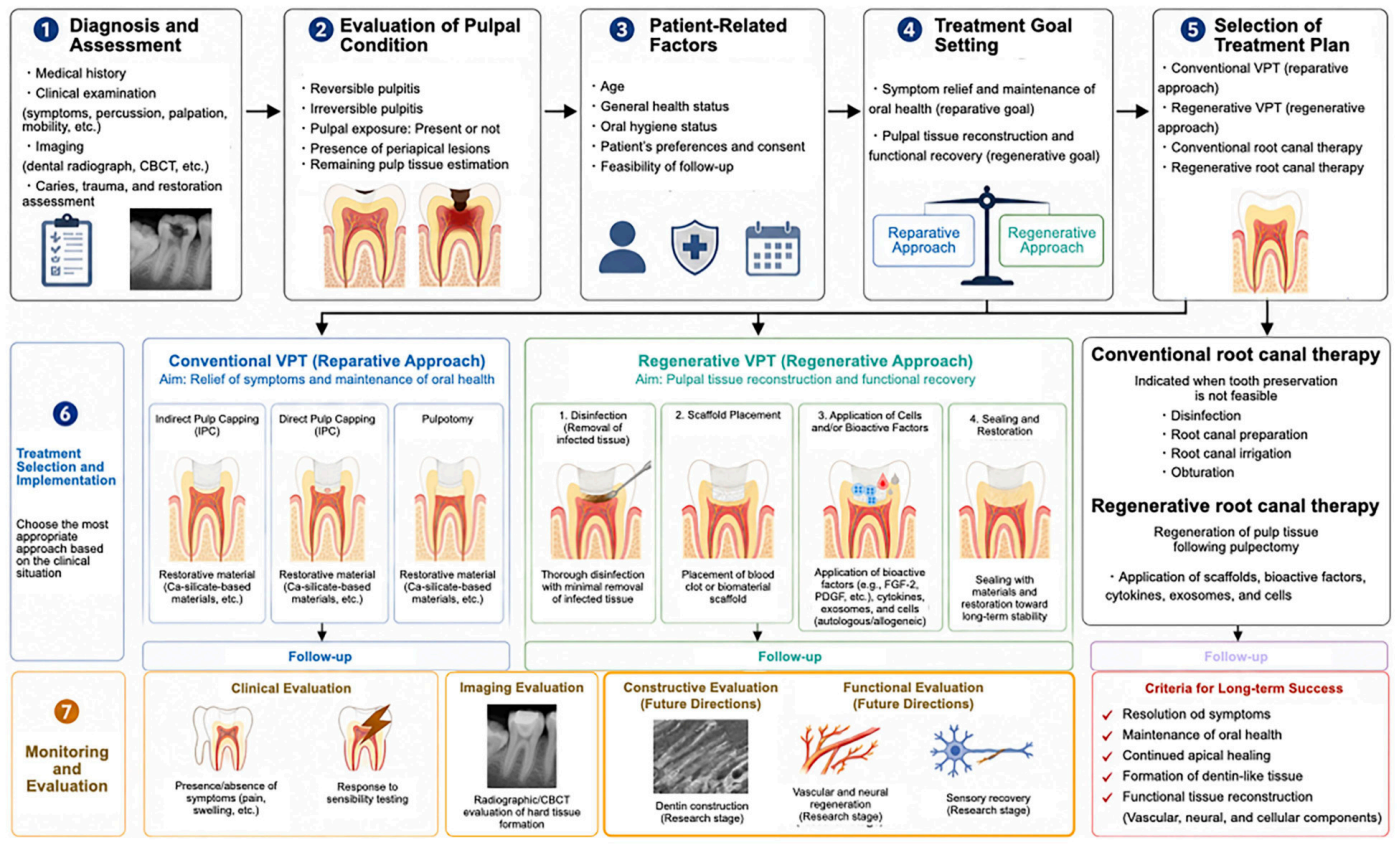
Clinical decision-making workflow for regenerative vital pulp therapy. This workflow illustrates a biologically stratified approach to regenerative vital pulp therapy. Clinical decision-making begins with assessment of pulpal symptoms, bleeding control, radiographic findings, residual pulp viability, and infection status. Mild inflammation with sufficient residual pulp vitality may be managed using conventional VPT with materials, whereas moderate inflammation or partial tissue loss may require scaffold- or signaling molecule-assisted regenerative VPT. More advanced tissue loss may require cell-based or tissue-engineering approaches to reconstruct vascularized and innervated pulp-like tissue. In immature teeth with pulp necrosis, regenerative endodontic procedures may support tissue in growth and continued root development. Across all stages, infection control, high-quality coronal sealing, caries and periodontal management, and long-term maintenance are essential for sustaining regenerative outcomes.

Despite significant advances in regenerative endodontics, major challenges remain in defining, evaluating, and clinically translating true dentin-pulp complex regeneration. This mini-review integrates the biological basis, biomaterial design, signaling systems, and clinical translational perspectives of dentin-pulp complex regeneration within a unified conceptual framework. Unlike previous reviews that have largely addressed individual components of regenerative endodontics, this review emphasizes the distinction between repair and functional regeneration and proposes conceptual criteria for evaluating regenerative outcomes. The integrated strategy for dentin-pulp complex regeneration and the clinical decision-making workflow for regenerative vital pulp therapy are summarized in Figures 1 and 2.

## 2 Biological basis of dentin-pulp complex regeneration

To distinguish repair responses from true D-P complex regeneration, proposed conceptual criteria are summarized in Table 1. This framework was conceptually developed based on previous descriptions of pulp healing and regenerative endodontic outcomes reported in the literature (14–16), while integrating structural, functional, and molecular perspectives into a translational classification system. These criteria were developed to conceptually distinguish different levels of regenerative outcomes in the D-P complex (14). The D-P complex is a structurally and functionally integrated tissue system responsible for tooth vitality, immune defense, and sensory function.

**Table 1:** Conceptual framework and proposed criteria for dentin-pulp complex regeneration.

Regenerative level	Dentin-related structural characteristics	Pulp-related findings	Structural parameters	Functional parameters	Molecular/biological parameters	Interpretation
Repair	Irregular tertiary/reparative dentin or calcified tissue formed predominantly within the pulp chamber; absence of physiological tubular architecture	Residual pulp tissue survival with fibrosis and/or persistent inflammation	Atubular or disorganized mineralized tissue; disrupted odontoblast layer continuity	Limited preservation of pulp vitality without restoration of physiological function	Predominance of inflammatory mediators; limited odontogenic marker expression	Healing response without restoration of original dentin-pulp structure or function
Partial regeneration	Partial reconstruction of dentin-like tissue at the defect site with incomplete tubular organization and limited structural continuity	Partial pulp tissue reconstruction with localized vascularization and reduced inflammation	Incomplete tubular dentin formation; partial extracellular matrix organization	Partial vascularization and/or sensory responsiveness	Expression of selected odontogenic and angiogenic markers (e.g., DSPP, DMP-1, VEGF)	Incomplete biological reconstruction with partial restoration of tissue components
Functional regeneration	Reconstruction of organized tubular dentin at the original defect site, closely resembling native dentin architecture and continuity	Regenerated pulp-like tissue with vascularization, innervation, immune regulation, and odontoblast-like cell layer formation	Continuous tubular dentin, organized odontoblast-like layer, physiologic extracellular matrix architecture	Functional vascular perfusion, neural responsiveness, immune competence, and physiological dentin formation capacity	Coordinated expression of odontogenic, angiogenic, neurogenic, and immunomodulatory markers	Restoration of both structural architecture and biological function of the dentin-pulp complex

Regeneration of the D-P complex is a multifaceted biological process that requires the coordinated reconstruction of multiple tissue components in a spatially and temporally regulated manner (15). True regeneration necessitates the restoration of the original architecture and function of the D-P unit. This process involves a cascade of biological events, including stem/progenitor cell recruitment, proliferation, lineage-specific differentiation, ECM deposition, angiogenesis, neurogenesis, and subsequent tissue remodeling. These events are tightly regulated by signaling pathways and the local microenvironment, often referred to as the stem cell niche (17, 18).

Among the various cell populations involved, dental pulp stem cells (DPSCs), first identified by Gronthos et al. (16), have been extensively investigated due to their high regenerative potential. DPSCs and SCAPs are major stem/progenitor cell populations involved in D-P regeneration due to their odontogenic differentiation capacity and regenerative potential. Other dental-derived stem cell populations may also contribute depending on the clinical context (19–23).

Recent advances in tissue engineering have enabled the development of strategies that more closely recapitulate the hierarchical and functional organization of the D-P complex. For instance, cell sheet engineering preserves cell-cell junctions, endogenous ECM, and growth factors, thereby facilitating the formation of more organized and biomimetic tissue structures (24, 25). In parallel, 3D constructs combining stem cells with scaffolds—such as hydrogels, bioceramics-containing matrices, and gelatin-based scaffolds—have shown promising results in promoting spatially controlled regeneration (4, 11–13). These scaffolds provide mechanical support and serve as delivery systems for bioactive molecules, highlighting the importance of scaffold design in regenerative outcomes.

Angiogenesis is a critical determinant of successful tissue regeneration, as the survival and function of newly formed tissue depend on adequate vascularization. Vascular endothelial growth factor plays a central role in promoting endothelial cell migration, proliferation, and new vessel formation. Inadequate vascular supply can lead to necrosis of regenerated tissue, underscoring the need for rapid and functional vascular network formation. Emerging strategies such as prevascularization and co-culture systems have been proposed to enhance angiogenic potential in engineered constructs (26).

Neurogenesis is essential for restoring pulp sensory function and is linked to angiogenesis (27, 28). This coordinated development is particularly important for achieving fully functional regeneration rather than mere structural repair.

Moreover, immune modulation has emerged as a key regulatory factor in D-P regeneration. The dental pulp possesses an intrinsic immune system that responds to injury and infection through innate and adaptive mechanisms. Macrophages, in particular, play a pivotal role through their ability to polarize into pro-inflammatory (M1) or anti-inflammatory/regenerative (M2) phenotypes. The balance between these phenotypes significantly influences healing outcomes. A pro-inflammatory environment may inhibit regeneration, whereas a controlled, pro-healing immune response can promote tissue wound healing, repair, and regeneration (29, 30). Recent evidence further suggests that repair and regeneration should not necessarily be regarded as completely separate biological events, but rather as a continuum influenced by the inflammatory microenvironment and the balance between destructive and pro-healing immune responses (16). In addition, cytokines, chemokines, and other inflammatory mediators dynamically shape the regenerative microenvironment, further emphasizing the importance of immunological regulation in regenerative endodontics.

Overall, regeneration of the D-P complex is a highly integrated process primarily driven by dynamic interactions among multiple cell populations, including stem/progenitor cells, vascular cells, neural elements, and immune cells. A deeper understanding of these cellular interactions is essential for advancing regenerative strategies that aim not only to achieve tissue repair but also to restore the structural and functional integrity of the D-P complex.

## 3 Role of growth factors and signaling molecules

Growth factors are essential regulators of tissue regeneration, regulating key cellular processes such as migration, proliferation, differentiation, and angiogenesis. In the context of D-P complex regeneration, these signaling molecules play pivotal roles in directing stem/progenitor cell behavior and coordinating tissue reconstruction. Among various signaling molecules investigated in regenerative endodontics, FGF-2 represents one promising strategy with relatively extensive translational evidence. FGF-2 promotes pulp tissue regeneration and dentin formation through activation of intracellular signaling pathways, including MAPK and PI3 K/Akt pathways (12, 13, 31). In addition, FGF-2 has been reported to influence ECM production and cellular recruitment, further contributing to tissue repair processes (32). Importantly, the mode of growth factor delivery critically influences regenerative outcomes. Controlled-release systems employing biodegradable carriers, such as hydrogels or gelatin-based scaffolds, enable sustained and localized delivery of FGF-2, resulting in improved tissue organization and more predictable regenerative responses (33). In contrast, rapid or uncontrolled release may lead to excessive or disorganized mineralization, often characterized by the formation of reparative dentin lacking proper tubular architecture (12).

Other growth factors, including bone morphogenetic proteins (BMP)-2, BMP-7, and vascular endothelial growth factor, play complementary roles in odontogenic differentiation and vascularization (34–36). Synergistic effects of multiple growth factors may be essential for functional regeneration (37). In addition, increasing attention has been directed toward Wnt signaling pathways and bioactive molecules released from the dentin matrix, both of which may contribute to endogenous regenerative responses by modulating stem/progenitor cell behavior and odontogenic differentiation (38). These nano-sized vesicles transport bioactive molecules, including proteins, lipids, and microRNAs, and have demonstrated promising potential as cell-free therapeutic tools in regenerative endodontics (39).

## 4 Biomaterials and scaffold design

Biomaterials are critical components in regenerative VPT, as they provide both structural support and biochemical cues necessary for tissue regeneration. Ideal scaffolds should provide biocompatible structural support while facilitating cell survival and tissue organization (40, 41).

Traditional materials such as calcium hydroxide and mineral trioxide aggregate (MTA) have been widely used in VPT due to their favorable biocompatibility and ability to induce dentin formation. However, these materials primarily promote reparative dentinogenesis characterized by irregular structure, rather than true regeneration of the D-P complex.

Recent advances have shifted attention toward bioactive scaffolds capable of modulating cellular behavior at the molecular level. Collagen-based scaffolds are among the most widely utilized due to their structural similarity to the natural ECM and their ability to support cell attachment and differentiation (42). In parallel, bioactive glass-containing materials have gained increasing interest due to their capacity to release therapeutic ions, such as calcium and silicon, which stimulate cellular activity, angiogenesis, and mineralization (43).

The integration of scaffolds with growth factors has further enhanced regenerative outcomes. These scaffold-assisted tissue engineering strategies provide a 3D microenvironment that supports residual pulp cells and facilitates organized tissue formation (44, 45). Controlled-release systems incorporated within scaffolds allow for sustained delivery of bioactive molecules, thereby improving spatial and temporal regulation of regeneration (46).

Emerging biomaterials, including hyaluronic acid-based scaffolds, offer additional advantages such as anti-inflammatory effects, enhanced cell migration, and improved wound healing responses (47). 3D bioprinting enables precise spatial control of cells and biomaterials. These technologies are particularly promising for recreating the hierarchical and functional architecture of the D-P complex (48).

## 5 From repair to regeneration: current challenges

Despite significant progress, several challenges remain in achieving predictable D-P regeneration. One of the major issues is the difficulty in clearly distinguishing between reparative dentin formation and true biological regeneration (49). While histological analysis remains the gold standard for evaluating tissue regeneration, its application in clinical settings is highly limited due to ethical and practical constraints (5). Most currently available clinical studies remain limited by small sample sizes, heterogeneous protocols, short follow-up periods, and the absence of standardized histological outcome assessment. Furthermore, ethical limitations restrict direct histological confirmation in humans, making interpretation of clinical success challenging.

Currently, clinical evaluation relies largely on indirect indicators, such as radiographic findings and pulp vitality tests (15). However, these methods provide only limited information regarding the structural organization and functional integration of regenerated tissues, making it difficult to accurately assess treatment outcomes. Importantly, conventional indicators of clinical success, such as pain relief, pulp vitality, and radiographic hard tissue formation, do not necessarily indicate true biological regeneration. In many cases, these outcomes may reflect successful repair or partial healing responses rather than complete restoration of the D-P complex. The site and organization of mineralized tissue formation should therefore also be considered when distinguishing repair from true regeneration. The lack of reliable, non-invasive diagnostic tools further complicates clinical decision-making and long-term evaluation.

Another critical challenge is the heterogeneity of regenerative outcomes. Patient-related factors, including age, systemic conditions, extent of inflammation, and the status of residual pulp tissue, can significantly influence treatment success and lead to variability in clinical results (15). Moreover, the local microenvironment, including bacterial load and immune response, may also affect the regenerative process.

In addition, regenerated tissues are often immature and frequently lack the highly organized tubular architecture characteristic of natural dentin (49). As a result, achieving complete structural and functional restoration remains a central goal in regenerative endodontics. To address these limitations, there is an urgent need to establish standardized criteria for defining and evaluating regeneration. Such criteria should integrate structural, functional, and molecular parameters rather than relying solely on mineralized tissue formation or pulp survival. Establishing standardized conceptual and clinical criteria for D-P complex regeneration will be essential for improving comparability among studies and facilitating clinical translation.

## 6 Clinical translation of regenerative VPT: human evidence

### 6.1 Clinical outcomes of conventional VPT

Recent studies have demonstrated that conventional VPT using calcium silicate–based materials achieves high clinical success rates even in mature permanent teeth and in cases diagnosed with irreversible pulpitis (50–52). However, these outcomes are primarily based on clinical indicators such as symptom resolution and maintenance of pulp vitality, and do not necessarily reflect true regeneration of the D-P complex.

### 6.2 Emerging regenerative approaches in human studies

Emerging regenerative approaches aim to reconstruct pulp-like tissue containing vascular, neural, and odontoblast-like components, and proof-of-concept has been demonstrated in human clinical studies (53, 54). Clinical investigations using autologous dental pulp stem cell transplantation and stem cells derived from exfoliated deciduous teeth have reported the formation of vascularized and innervated pulp-like tissue, and recent studies have further suggested the feasibility of applying such approaches to mature teeth (53). Importantly, most currently reported clinical regenerative strategies are not true “regenerative VPT” procedures, but instead involve pulp tissue regeneration following pulpectomy, highlighting a gap between the conceptual framework of regenerative VPT and its current clinical implementation.

From a clinical perspective, conventional VPT and regenerative approaches should not be regarded as competing strategies, but rather as complementary options positioned along a biological continuum. While conventional VPT is relatively simple and supported by a substantial body of clinical evidence, regenerative approaches offer greater biological potential but are associated with technical complexity, cost, and challenges in standardization.

### 6.3 Current limitations and translational challenges

However, direct comparative clinical studies between these approaches remain limited, and there is currently insufficient evidence to conclusively demonstrate that regenerative strategies provide superior long-term functional outcomes—including vascular and neural restoration—compared with conventional VPT (55, 56). Furthermore, current clinical outcome measures are not directly comparable between the two approaches: conventional VPT is primarily evaluated using survival-based endpoints, such as maintenance of pulp vitality, whereas regenerative approaches aim to achieve functional tissue restoration, making direct comparison inherently challenging.

Although early human studies have demonstrated proof-of-concept for pulp tissue regeneration, the currently available evidence remains limited by small sample sizes, heterogeneous methodologies, and insufficient long-term functional evaluation. Therefore, further well-designed clinical studies with standardized outcome criteria are required to clarify the long-term translational value of regenerative approaches.

## 7 Discussion and future perspectives

The regeneration of the D-P complex represents a major paradigm shift in endodontic treatment, moving from passive repair toward biologically driven reconstruction. Conventional endodontic therapies have primarily focused on infection control and structural preservation; however, they do not restore essential biological functions of the pulp, such as vascularization, innervation, and immune competence. In contrast, regenerative approaches aim to re-establish these functions and preserve the tooth as a living organ (5, 6).

Successful D-P regeneration requires precise control of the local microenvironment, including the coordinated regulation of stem/progenitor cell recruitment, proliferation, differentiation, angiogenesis, and neurogenesis. The development of a supportive regenerative niche is therefore critical. Advances in biomaterials, particularly bioactive scaffolds and controlled delivery systems, have enabled spatial and temporal regulation of bioactive signals, thereby promoting tissue organization and regeneration (44, 45, 47).

Despite these advances, several challenges remain for clinical translation. The oral cavity is a non-sterile environment, and reinfection due to secondary caries or microleakage remains a significant risk. Consequently, long-term success depends not only on tissue regeneration but also on continuous control of the oral environment, including caries management, periodontal care, reliable coronal sealing, and patient adherence. This dependency distinguishes D-P regeneration from regeneration in many other organs. Another key consideration is the biological heterogeneity of pulpal conditions. The degree of inflammation and residual tissue viability vary widely among cases, making a one-size-fits-all approach inappropriate. Previous studies have shown that VPT outcomes are strongly influenced by the severity of pulpal inflammation and the presence of viable pulp tissue (15). Therefore, regenerative strategies should be biologically stratified. Mild injuries may be managed with conservative VPT using bioactive materials, whereas more extensive tissue loss may require scaffold-assisted or cell-based approaches. In cases of complete pulp removal, successful regeneration likely requires the integrated application of stem cells, biomaterials, and signaling molecules. Therefore, treatment strategies should balance regenerative efficacy, reproducibility, patient burden, cost-effectiveness, and long-term stability.

Emerging technologies, including 3D bioprinting, offer unprecedented control over the spatial organization of cells and biomaterials, enabling reconstruction of complex tissue architectures (47). In addition, exosome-based therapies have gained attention as cell-free regenerative approaches (39), and advances in stem cell engineering may further enhance regenerative capacity and reproducibility. Future regenerative strategies may involve not only scaffold-based tissue engineering, but also endogenous cell homing, immunomodulatory approaches, extracellular vesicles, and biologically inspired minimally invasive therapies. Many regenerative approaches have shown promising results in preclinical studies, but their clinical translation is limited by variability in outcomes and lack of standardized protocols. To overcome these challenges, well-designed clinical trials with clearly defined endpoints are essential, along with the establishment of standardized evaluation criteria incorporating structural, functional, and molecular parameters. Ultimately, achieving predictable and functional D-P regeneration will require an integrated, multidisciplinary approach combining advances in biology, biomaterials science, and clinical practice. By adopting biologically stratified strategies and leveraging emerging technologies, next-generation therapies may ultimately enable not only tooth preservation but also restoration of more complete biological function. At the same time, long-term success fundamentally depends on preventive strategies and sustained management of the oral environment.
